# Suitability of Three Trunk Traps for Capturing Larvae of *Lymantria dispar* (L.) (Lepidoptera, Erebidae)

**DOI:** 10.3390/insects16050522

**Published:** 2025-05-15

**Authors:** Tanja Bohinc, Paraskevi Agrafioti, Stelios Vasilopoulos, Evagelia Lampiri, Maria C. Boukouvala, Anna Skourti, Demeter Lorentha S. Gidari, Nickolas G. Kavallieratos, Xavier Pons, Alexandre Levi-Mourao, Elena Domínguez Solera, Enrique Benavent Fernandez, Anna Roig Pinãs, Christos G. Athanassiou, Stanislav Trdan

**Affiliations:** 1 Department of Agronomy, Biotechnical Faculty, University of Ljubljana, 1000 Ljubljana, Slovenia; tanja.bohinc@bf.uni-lj.si; 2 Laboratory of Entomology and Agricultural Zoology, Department of Agriculture, Crop Production and Rural Environment, University of Thessaly, 382 21 Nea Ionia, Greece; agrafiot@uth.gr (P.A.); svasilopoulos@gmail.com (S.V.); elampiri@uth.gr (E.L.); athanassiou@uth.gr (C.G.A.); 3 Laboratory of Agricultural Zoology and Entomology, Department of Crop Science, Agricultural University of Athens, 118 55 Athens, Greece; mbouk@aua.gr (M.C.B.); annaskourti@aua.gr (A.S.); dimitralorentha@gmail.com (D.L.S.G.); nick_kaval@aua.gr (N.G.K.); 4 Department of Crop and Forest Sciences, Agrotechnio Centre, Universitat de Lleida, 25003 Lleida, Spain; xavier.pons@udl.cat (X.P.); alexandrelevi.garcia@udl.cat (A.L.-M.); 5 AIMPLAS, Plastics Technology Centre, València Parc Tecnològic, 46980 Valencia, Spain; edominguez@aimplas.es (E.D.S.); ebenavent@aimplas.es (E.B.F.); 6 PROBODELT, Pest Control Company, 43870 Tarragona, Spain; anna@probodelt.com

**Keywords:** spongy moth, caterpillars, trap devices, suitability, forest, urban area

## Abstract

In this paper, we present the results of field investigations on the suitability of three types of trunk traps (‘Commercial 1’, ‘Commercial 2’, and ‘Prototype’) for capturing spongy moth (*Lymantria dispar*) larvae in two areas with different climates and forest ecosystems. In areas of NE Slovenia, which were characterized by Pannonian climate and regular rainfall during the research period (2022–2024), the ‘Commercial 2’ trap was the most suitable for the capture of old larvae. In a forest with 20–30 m tall trees with the dominant species *Quercus robur*, *Carpinus betulus*, and *Pinus sylvestris*, it proved easy to set ‘Commercial 2’ on trunks and change their parts upon inspection for captures. ‘Commercial 1’ traps proved to be less suitable, since their bags quickly were filled with water during downpours and consequently fell out of the traps. ‘Prototype’ traps proved to be the most suitable in an area with a Mediterranean climate (Greece), where the trees (*Quercus* spp.) were smaller (3–4 m) and both younger and older larvae could more easily come into contact with the sticky surface of the inner part of the trap, of which longer-lasting effectiveness was possible in a drier climate. For the mass trapping of larvae in urban areas with a more humid climate, we therefore suggest the use of ‘Commercial 2’ traps, and in areas with a drier climate, the use of ‘Prototype’ traps.

## Simple Summary

The suitability of three different types of trunk traps, ‘Commercial 1’, ‘Commercial 2’, and ‘Prototype’, for capturing spongy month larvae was evaluated in Slovenia and Greece. During the period 2022–2024, we deployed traps in forests in northeastern Slovenia. The trunk trap ‘Commercial 2’ was the most suitable trap for the capture of spongy moth larvae in Slovenian forests. During the period 2023–2024, ‘Prototype’ traps were the most efficient in a Greek forest in Ileia Prefecture (Peloponnese). We concluded that the different efficacy was mostly detected based on different climates and forest ecosystems. ‘Commercial 1’ and ‘Commercial 2’ were suitable for the Slovenian (humid) climate, while ‘Prototype’ was suitable for dry Mediterranean climate in Greek areas.

## 1. Introduction

The spongy moth, *Lymantria dispar* (L.) (Lepidoptera: Erebidae) is primarily a pest in forests, the larvae of which cause baldness or defoliation of various species of deciduous trees and some conifers when they appear in high numbers. During outbreaks, which usually last 3–4 years, the caterpillars often feed on the buds and leaves of fruit trees. Although the spongy moth is a polyphagous insect species, it shows a preference for certain tree species, such as oak (*Quercus* spp.), European hornbeam (*Carpinus betulus*), European hop-hornbeam (*Ostrya carpinifolia*), beech (*Fagus* spp.), hazel (*Corylus* spp.), and alder (*Alnus* spp.) [1,2].

In regions where it has been introduced, such as North America, outbreaks occur more frequently than in its native range of Europe and Asia [3,4]. Numerous reports on the results of attempts to control the spongy moth in Europe confirm the fact that its natural enemies are not effective. This means that synthetic [5], plant [6], and biological insecticides [7] are used to reduce the economic importance in all areas of its distribution. It has been documented [8] that the use of synthetic insecticides reduces the effectiveness of native natural enemies of the spongy moth.

Urban environments, where forest trees play a vital role in green infrastructure, are particularly affected by spongy moth caterpillars [9,10]. Early-instar larvae (typically first- to third-instar) feed on host leaves during the day and stay on the underside of foliage during the evening. Late-instar larvae (fourth- to sixth-instar) feed on the canopy overnight, whereas at daybreak, they move downward in search of cryptic resting places (e.g., bark flaps, bark crevices, or litter under the host tree), as these sites provide protection from predators [1,11]. In this way, larvae come into contact with park users, causing serious allergies (dermatitis) [12,13].

The use of insecticides is becoming less desirable or even prohibited in urban environments [14], so experts are studying environmentally acceptable ways to effectively capture the spongy moth larvae. Despite the economic importance of the spongy moth in many areas of the northern hemisphere, we have the results of only one study (USA) examining the effect of sticky trunk barrier on caterpillars [15,16], and in Europe, mechanical traps for catching caterpillars were not used before our research. Our research includes the first trial with specific trunk traps for spongy moth caterpillars in Europe, the results of which will be useful especially in urban environments. The importance of the results is even greater because they come from two climatically quite different areas of Europe, and the weather can significantly affect the suitability (efficiency) of traps.

Environmentally friendly pest control methods also include the use of mechanical methods, which have increasing potential due to the increasing restrictions on the use of chemical products in plant protection [17,18]. In our study, we investigated the suitability of three types of mechanical trunk traps for trapping spongy moth larvae, which can be important causative agents of dermatitis in humans when occurring in large numbers in urban environments [13]. Two of the trap types used in our study have been used quite effectively to pine processionary moth (*Thaumetopoea pityocampa* [Denis and Schiffermüller]) larvae in previous years [18], and one of the aims of our investigation was to extend their use for the capture of the spongy moth larvae. According to [16,19], sticky trunk barriers have been a good solution to restrict the movement of spongy moth larvae up to the boles of trees.

In Slovenia, the last outbreak of this pest was recorded in 2004 in the western part of the country [20], while in Greece, higher abundances of spongy moth are much more common; the last outbreak was thus recorded in the period 2016–2017 [1]. It is known that tree species composition is an important factor of insect outbreaks [21]. The tree species that are represented in the areas of Slovenia and Greece, where we conducted research in the period 2022–2024, are suitable hosts for spongy moth. Among the most suitable hosts are different species of oak and European hornbeam [22,23].

In some countries, such as Slovenia and Greece, pests of forest trees are usually not controlled systematically, and this is often the case even if they occur in large numbers on trees in an urban environment. An important reason for this fact is the negative opinion of residents of urban areas about chemical protection of plants, even though some of such methods (for example, endotherapeutic methods of insecticide application) are human- and environment-friendly [24,25]

In this paper, we present the results of a study evaluating the effectiveness and suitability of three different types of mechanical trunk traps for capturing spongy moth larvae. The study examines trap design, capture efficiency, and potential applications in integrated pest management strategies for controlling spongy moth populations.

## 2. Materials and Methods

### 2.1. Timeframe and Trap Deployment Areas

Mechanical trunk traps for capturing spongy moth larvae were deployed in Slovenia and Greece. In Slovenia, trunk traps were set in the northeastern part of the country, in the Ginjevec (46.620706, 16.352949), and Murska šuma (46.514198, 16.520675) forests, in the period 2022–2024. Both sites are located in an area with a Pannonian climate. The Ginjevec forest covers 380 ha, and the most common tree species are English oak (*Quercus robur*), European hornbeam (*Carpinus betulus*), Scots pine (*Pinus sylvestris*), black alder (*Alnus glutinosa*), European ash (*Fraxinus excelsior*), and field maple (*Acer campestre*). The most common tree species found in Murska šuma are English oak, ash, hornbeam, black alder, poplar (*Populus* spp.), willow (*Salix* spp.), and black walnut (*Juglans nigra*). In the Ginjevec forest, two areas were designed for the purpose of the survey, namely ‘Ginjevec 1’ as Area 1 and ‘Ginjevec 2’ as Area 2. Area 1 (0.55 ha) and Area 2 (0.60 ha) were located 2 km apart from each other. Area 3 (0.8 ha) was located in Murska šuma, which is 25 km away from the Ginjevec forest. Murska šuma has an area of 100 ha. The average height of trees in all three areas ranged from 20 to 30 m, and the average density was 100 trees/ha. Ginjevec is a state forest, while Murska šuma is privately owned forest. Permission was obtained from the owners to carry out the experiments.

In Greece, the mechanical trunk traps were deployed in the Ileia Prefecture (Peloponnese) in three areas: Area 1 (Kryoneri cemetery; 0.71 ha, 37.46198, 21.79991), covering an area of 7123 ha; Area 2 (Petralona Trianda Road 2; 0.76 ha, 37.43859, 21.82632), covering an area of 7570 ha; and Area 3 (Petralona; 0.63 ha, 37.44109, 21.82849), covering an area of 6253 ha. All three areas are characterized by Mediterranean climate. The most frequent tree species are oaks (*Quercus* spp.). The average tree size in all three areas ranged from 3 to 4 m, and the average density was 100 trees/ha. The study was performed in the years 2023 and 2024.

### 2.2. Trap Designs

Three different types of mechanical trunk traps were included in the experiment, namely (1) the Econex type (hereafter ‘Commercial 1’) produced by Sanidad Agricola Econex (Murcia, Spain), (2) the SanSan type (hereafter ‘Commercial 2’) produced by SanSan Prodesing SL (Náquera-Valencia, Spain), and (3) a prototype (hereafter ‘Prototype’) produced in the framework of the present project by Aimplas (Paterna-Valencia, Spain).

**Figure 1:**
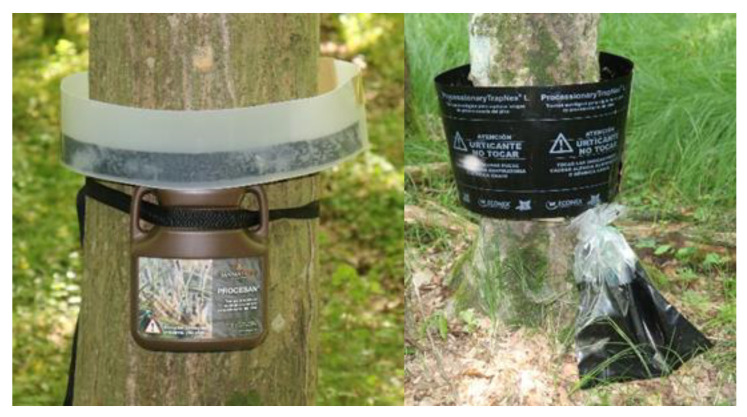
‘Commercial 2’ (**left**) and ‘Commercial 1’ (**right**) mechanical trunk traps in the Ginjevec forest (photo: S. Trdan).

The ‘Commercial 1’ mechanical trunk trap (Figure 1, right) consisted of a sponge foam (polyurethane foam) (dimensions 150 × 7 × 5 cm) that fitted over the trunk of the tree. On the outside of the foam, a black PE barrier (dimensions 150 × 25 × cm and 0.3 mm thick) was placed, which was sticky on the inside. A 15 cm hose was placed through a round hole (2 cm) in the foam, and a plastic bag (6 L) was placed on the underside of the foam to capture larvae. The mechanical trap is known commercially as ProcessionaryTrapNex^®^ L and is designed to trap pine processionary moth larvae.

The ‘Commercial 2’ mechanical trunk trap (Figure 1, left) consisted of a sponge foam (polyurethane foam) (200 × 4.5 × 4 cm) that fitted over the trunk of the tree. A clear barrier made of stronger plastic (200 × 14 × 0.1 cm) was attached to the outside of the sponge foam with adhesive tape, and a brown plastic container (23 × 13 × 34 cm or 2.4 L) was placed on the underside of the foam to trap larvae. The plastic container was attached to the trunk with a connecting strap. The trunk trap is commercially known as Procesan^®^ and is designed to trap pine processionary moth larvae.

**Figure 2:**
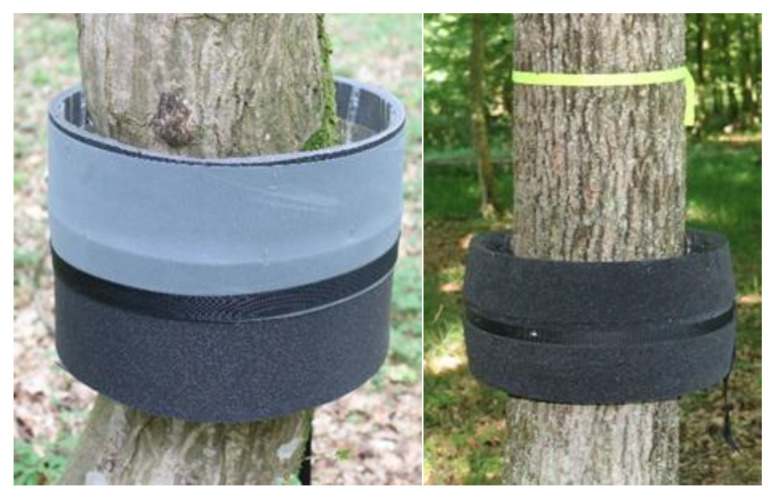
‘Prototype’ mechanical trunk trap, deployed in 2023 (**left**) and 2024 (**right**) in the Ginjevec forest (photo: S. Trdan).

**Figure 3:**
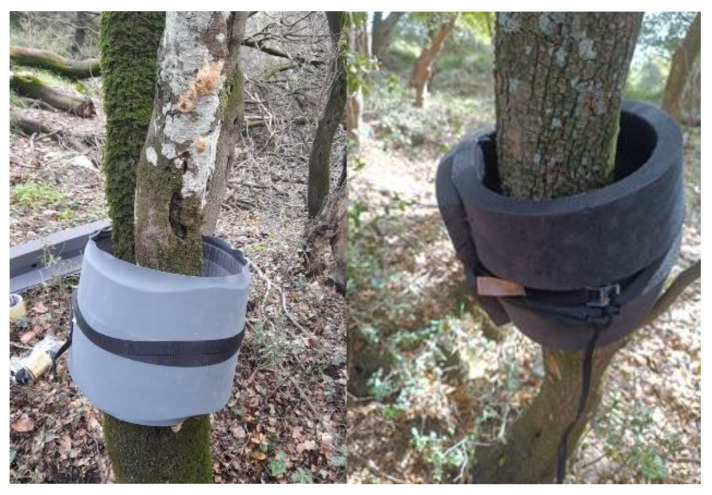
‘Prototype’ mechanical trunk trap, deployed in 2023 (**left**) and 2024 (**right**) in Greece (photo: M. C. Boukouvala and A. Skourti).

The ‘Prototype’ trap (Figure 2 and Figure 3), manufactured at Aimplas, was studied in both countries in 2023 and 2024. The ‘Prototype’ trap consisted of two parts, an inner part that encircled the trunk (7 × 8 cm) and an outer part (25 × 0.6 cm in 2023; 25 × 2.5 cm in 2024) 8 cm away from the trunk that was placed on the outer side of the inner part of the trap, parallel to the trunk. A double-sided adhesive tape was placed on the inner side of the outer part of the trap, and its adhesive strength was checked at each trap inspection and, if necessary, strengthened by applying adhesive (tangle trap; manufacturer: Andermatt Biocontrol, Grossdietwil, Switzerland; representative for Slovenia: Metrob Ltd., Začret, Slovenia). In 2024, the ‘Prototype’ trap included felt instead of double-sided sticky tape, on which the above-mentioned adhesive was applied. In Slovenia, approximately 30 g of adhesive was applied to each prototype trap when necessary. TEMO-O-CID adhesive (producer: Adama Ltd., Raleigh, NC, USA) was used to set the ‘Prototype’ traps at the Greek sites.

### 2.3. Deployment and Monitoring

In all years of the study, except for 2024 in Slovenia, trunk traps were set on tree trunks under spongy moth egg clusters. Depending on the height of the egg clusters, trunk traps were set at a height of 0.5 to 1.5 m from the ground. In Slovenia, the experiment was carried out in three blocks within each area (mentioned under Section 2.1). Each block consisted of two traps of each type (‘Commercial 1’, ‘Commercial 2’, and ‘Prototype’) for a total of six traps. There were three blocks per area for a total of 18 traps. In Slovenia, in 2024, trunk traps were set on trunks without egg clusters because we wanted to test the capture of larvae in traps under such conditions.

Greek experimental design was based on two blocks per specific area. Each block consisted of three traps (one ‘Commercial 1’, one ‘Commercial 2’, and one ‘Prototype’. The study was performed in three areas.

**Figure 4:**
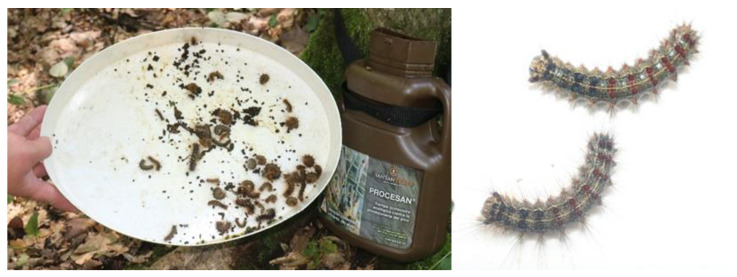
Captures of old spongy moth larvae in a ‘Commercial 2’ trunk trap (**left**); old larvae (**right**) (photo: S. Trdan).

In Slovenia, mechanical trunk traps were set on 5 May in 2022, on 20 April in 2023, and on 22 April in 2024. Larvae caught in the trunk traps were counted at 7- to 10-day intervals. In all three years of the study, we determined the abundance of older larvae, while the number of young larvae was determined only in 2023, when traps were placed early enough to intercept them. Larvae trapped in ‘Commercial 1’ and ‘Commercial 2’ traps were shaken on a light plastic surface before counting (Figure 4), while larvae caught in ‘Prototype’ traps were counted on a sticky surface. In all three years of the study, monitoring was carried out until mid-July (15 July in 2022, 19 July in 2023, and 10 July in 2024).

**Figure 5:**
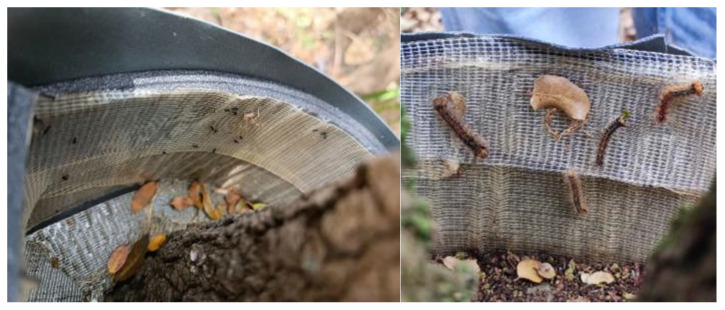
Captures of early- (**left**) and late-instar (**right**) spongy moth larvae in the ‘Prototype’ mechanical trunk trap in 2023 in Greece (photo: A. Skourti).

**Figure 6:**
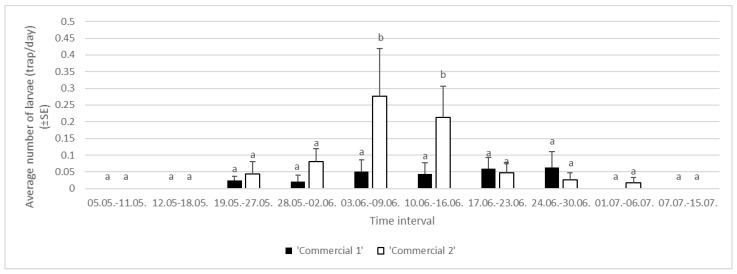
Average numbers of larvae of *Lymantria dispar* caught in trunk traps per day in specific time intervals in 2022 in three areas of Slovenia (lowercase letters represent significant differences within each area following specific time intervals: 19–27 May, F = 3.19, *p* = 0.2596; 28 May–2 June, F = 9.99, *p* = 0.1213; 3–9 June, F = 11.10, *p* < 0.05; 10–16 June, F = 9.15, *p* < 0.05; 17–23 June, F = 7.14, *p* = 0.6233; 24–30 June, F = 10.12, *p* = 0.4010; 1–6 July, F = 5.17, *p* = 0.0599).

**Figure 7:**
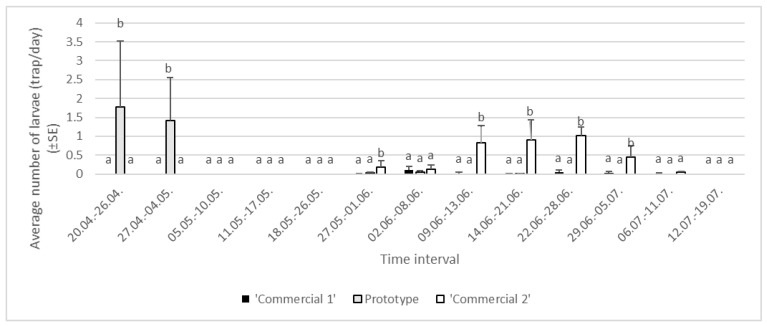
Average numbers of *Lymantria dispar* larvae caught in trunk traps per day in specific time intervals in 2023 in three areas of Slovenia (lowercase letters represent significant differences within each area following specific time intervals: 20–26 April, F = 12.08, *p* < 0.05; 27 April–4 May, F = 17.50, *p* < 0.05; 27 May–1 June, F = 16.14, *p* < 0.05; 2–8 June, F = 17.42, *p* = 0.1510; 9–13 June, F = 15.74, *p* < 0.05; 14–21 June, F = 9.03, *p* = 0.02; 22–28 June, F = 6.07, *p* < 0.05; 29 June–5 July, F = 17.17, *p* < 0.05; 6–11 July, F = 14.20, *p* = 0.0625).

**Figure 8:**
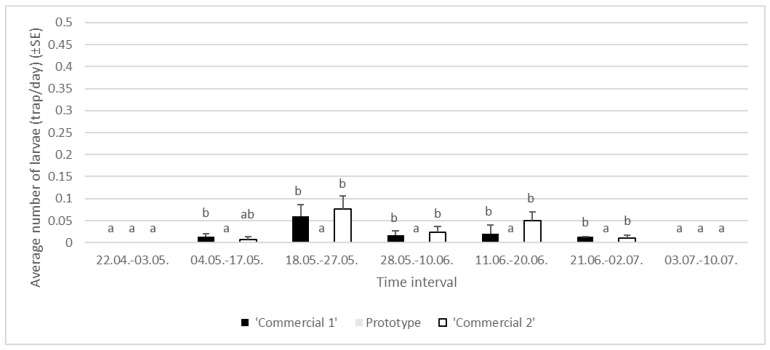
Average numbers of *Lymantria dispar* larvae in trunk traps per day in specific time intervals in 2024 in three areas of Slovenia (lowercase letters represent significant differences within each area following specific time intervals; for Area 1: 4–17 May, F = 19.07, *p* < 0.05; 18–27 May, F = 18.30, *p* < 0.05; 28 May–10 June, F = 9.06, *p* < 0.05; 11–20 June, F = 14.10, *p* < 0.05; 21 June–2 July, F = 13.13, *p* < 0.05).

**Figure 9:**
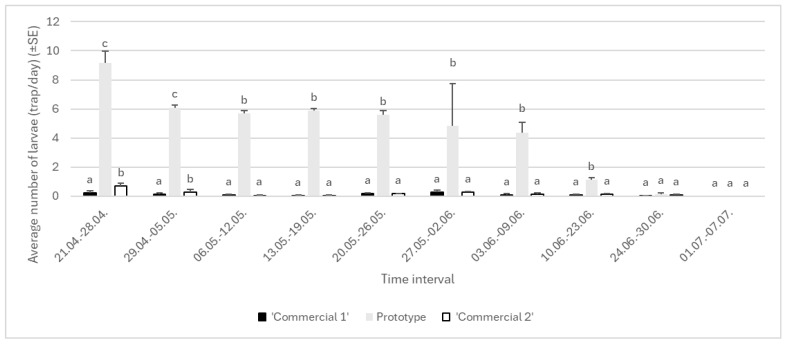
Average number of *Lymantria dispar* larvae caught in trunk traps per day in specific time interval in 2023 in three areas of Greece (lowercase letters represent significant differences within each area following specific time intervals; for Area 1: 21–28 April, F = 27.33, *p* < 0.05; 29 April–5 May, F = 24.13, *p* < 0.05; 6–12 May, F = 22.13, *p* < 0.05; 13–20 May, F = 30.99, *p* < 0.05; 21–26 May, F = 23, *p* < 0.05; 27 May–2 June, F = 24.11, *p* < 0.05; 3–9 June, F = 12.30, *p* < 0.05; 10–23 June, F = 27.02, *p* < 0.05; 24–30 June, F = 15.23, *p* = 0.0685).

**Figure 10:**
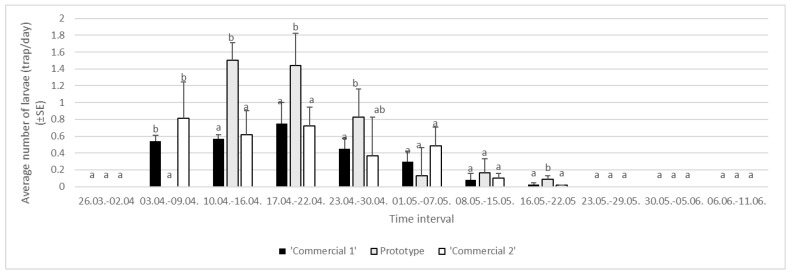
Average numbers of *Lymantria dispar* larvae caught in trunk traps per day in specific time intervals in 2024 in three areas of Greece (lowercase letters represent significant differences within each area following specific time intervals; for Area 1: 3–9 April, F = 15.12, *p* < 0.05; 10–16 April, F = 25.12, *p* < 0.05, 17–22 April, F = 21.13, *p* < 0.05; 23–30 April, F = 17.12, *p* < 0.05, 1–7 May, F = 20.16.13, *p* < 0.05; 8–15 May, F = 33.12, *p* < 0.05; 16–22 May, F = 22.13, *p* < 0.05).

In 2023, the monitoring of the effectiveness of the mechanical trunk traps in Greece was carried out from 21 April until 7 July (Figure 5), and in 2024 the mechanical trunk traps were set on 26 March. The trial ended on 11th June. In both years of the study, we determined the abundance of larvae without separating them into older and younger ones. The data are presented (Figure 6, Figure 7, Figure 8, Figure 9 and Figure 10) as average captures per trap per day over the selected time interval.

### 2.4. Weather Data

Environmental data, namely average daily temperature (°C) and precipitation (mm), were recorded to assess the influence of the weather. In Slovenia, the data were retrieved by the Slovenian Environment Agency’s Data Archive [26]. For Areas 1 and 2, the data were obtained by the Rakičan weather station, while those for Area 3 were obtained by the Lendava weather station. Therefore, data for Areas 1 and 2 are presented in the same columns in the tables from Results section. In Greece, the data were obtained by the Hellenic National Meteorological Service. Based on the data obtained, the average daily temperature and average daily precipitation were calculated over the selected time interval. Additionally, the total precipitation (mm) was determined for the entire period, and the number of rainfall days (rainy days) within the timeframe was recorded. A rainy day was defined as a day with more than 1 mm of rain.

The sum of effective temperatures was calculated (degree day computations, DD) for both countries. The temperature threshold (7 °C) was chosen based on the literature [27]. The DD value was calculated as the average daily temperature above 7 °C. The calculation started on 1 January in all years of the study. For Slovenia, in the presentation of the results, we highlight the DD values at the first and last captures of larvae. We also present the DD values at the time of the highest larvae captures. In the Greek data, we highlight the DD at the first and last larvae captures.

The DD was calculated as follows [28,29]:

EFT_daily_ = T_daily_ − 7 °C,

where EFT_daily_ is the daily effective temperature from the 1st of January of the year of observation until the last day of the experiment (trunk traps at the location), and T_daily_ is the average daily air temperature;

DD = ΣEFT_daily_,

where DD represents the sum of the effective temperatures and ΣEFT_daily_ represents the sum of the effective daily temperatures [28,29].

### 2.5. Statistical Analysis of Data

For the trunk trap comparison, multiple ANOVA (with year of experiment, location, and trap type as main effects and average number of larvae caught in trunk traps per trap per day as the response variable) was performed separately for each country. To determine differences within experimental years, two-way ANOVA was performed (with location and trap type as main effects and average number of larvae caught in trunk traps per trap per day as the response variable) to locate differences between trunk traps and areas. Before the analysis, counts were transformed to log (x + 1) to normalize variances and standardize means [30]. Means were separated by the Tukey HSD test at 0.05 probability. Statgraphics Centurion XIX [31] was used for the statistical analysis. Average daily temperatures and average daily precipitation values are presented as average values in specific time intervals. Sum of precipitation and number of rainy days are presented as sums per specific time interval.

## 3. Results

### 3.1. Slovenia

The average daily captures of larvae in trunk traps were significantly affected by trap type (df = 2, F = 10.24, *p* < 0.05). The average daily captures in traps also differed between years (df = 2, F = 24.14, *p* < 0.05), while it was not significantly affected by location (area) (df = 2, F = 0.86, *p* = 0.4257). No impact of experimental block was also detected (df = 2, F = 0.38, *p* = 0.6826). There were also no interactions detected between major factors, i.e., trap type and experimental year (df = 4, F = 3.33, *p* = 0.1210), experimental year and experimental area (df = 4, F = 15.80, *p* = 0.4592), or experimental area and trap type (df = 4, F = 7.15, *p* = 0.7345).

#### 3.1.1. Year 2022

Analysis of the 2022 data showed a significant effect of trunk trap type on the average daily captures of larvae (df = 1, F = 10.24, *p* < 0.05), while there was no significant effect of experimental area (df = 2, F = 8.88, *p* = 0.0622) or block (df = 2, F = 0.59, *p* = 0.0785) on the same response variable. We did not detect interactions between trap type and experimental area (df = 3, F = 4.12, *p* = 0.5943) or between trap type and experimental block (df = 3, F = 1.15, *p* = 0.8586). In 2022, the average captures in ‘Commercial 1’ traps were 0.02 larvae per trap per day, while ‘Commercial 2’ traps caught an average of 0.07 ± 0.01 larvae per trunk trap per day. The first trap captures were observed in the interval 19 May–27 May, when less than 0.1 larvae per trunk trap per day was found in the traps. The highest captures were observed in the interval 3–9 June, when more than 0.25 larvae/trap were detected in ‘Commercial 2’ traps. After 6 July, no larvae were detected in the tested trunk traps. All together, we caught 48 larvae in Area 1 (8 in ‘Commercial 1’ and 40 in ‘Commercial 2’ traps), 25 larvae in Area 2 (4 in ‘Commercial 1’ and 21 in ‘Commercial 2’ traps), and 46 larvae in Area 3 (17 in ‘Commercial 1’ and 29 in ‘Commercial 2’ traps) (Figure 6).

#### 3.1.2. Year 2023

In 2023, the number of caught larvae differed between different trunk trap types (df = 2, F = 4.70, *p* < 0.05) and experimental areas (df = 2, F = 2.35, *p* < 0.05). There was no impact of experimental block detected (df = 2, F = 7.05, *p* = 0.7222). No interactions between major project factors were detected, i.e., between trap type and experimental area (df = 4, F = 8.02, *p* = 0.9453) or between trap type and experimental block (df = 4, F = 9.12, *p* = 0.1418). The average captures of larvae were significantly the lowest in ‘Commercial 1’ trunk traps (0.03 ± 0.01 larvae/trap/day), while no significant differences were found between the average captures in ‘Commercial 2’ traps (0.29 ± 0.06 larvae/trap/day) and ‘Prototype’ traps (0.20 ± 0.09 larvae/trap/day). More than one young larva was detected in ‘Prototype’ traps within two-week intervals from the beginning of the season, where they were attached to the sticky layer. In the two commercial trap types, no larvae were detected during this period. Larvae that were detected in ‘Commercial 1’ and ‘Commercial 2’ traps from the period 27 May–11 July were evaluated as old larvae. In the period from 9 June to 5 July, the highest abundance of larvae was detected in ‘Commercial 2’ traps, more than 0.5 larvae/trap/day. In general, 1000 larvae were caught in this experimental year, 266 in Area 1 (8 in ‘Commercial 1’, 157 in ‘Prototype’, and 101 in ‘Commercial 2’ traps), 599 in Area 2 (32 in ‘Commercial 1’, 113 in ‘Prototype’ and 309 in ‘Commercial 2’ traps), and 135 in Area 3 (2 in ‘Commercial 1’, 76 in ‘Prototype’, and 57 in ‘Commercial 2’ traps) (Figure 7).

#### 3.1.3. Year 2024

In 2024, the number of caught larvae did not differ between different trunk trap types (df = 2, F = 10.67, *p* = 0.0755) or among the three areas under investigation (df = 2, F = 2.35, *p* = 0.0958). No impact of experimental block was detected (df = 2, F = 12.11, *p* = 0.8345). No interactions between major factors were detected, i.e., between trap type and experimental area (df = 4, F = 9.99, *p* = 0.1115) or between trap type and experimental block (df = 4, F = 4.87, *p* = 0.2117).

On average, we found 0.02 ± 0.01 larvae/trap/day in ‘Commercial 1’ traps and 0.03 ± 0.01 larvae/trap/day in ‘Commercial 2’ traps, while no captures were recorded in ‘Prototype’ traps (Figure 8). In general, 24 old larvae were detected in Area 1 (10 in ‘Commercial 1’ and 14 in ‘Commercial 2’ traps). In Area 2, we caught 10 old larvae. None of them were caught in ‘Prototype’ traps. In Area 3, we caught 5 old larvae, only in ‘Commercial 1’ traps. The highest average number of larvae was detected in ‘Commercial 1’ and ‘Commercial 2’ traps in the period 18 May–27 May, when more than 0.05 larvae/day was caught in both trap types.

In all years, ‘Commercial 2’ traps proved to be most effective, capturing between 58.3% (2024) and 90.2% (2023) of all older larvae. The higher number of young larvae in the ‘Prototype’ traps in 2023 accounted for 40.5% of the larvae captures overall.

### 3.2. Greece

The mean daily capture of larvae in traps was significantly influenced by trunk trap type (df = 2, F = 17.13, *p* < 0.05) and year of survey (df = 1, F = 10.11, *p* < 0.05), while experimental location had no significant effect on the average daily captures of larvae (df = 2, F = 2.22, *p* = 0.3333). No experimental block was detected (df = 1, F = 10.13, *p* = 0.4256). There was no significant impact of interactions detected, i.e., between trap type and experimental area (df = 4, F = 16.12, *p* = 0.7421).

#### 3.2.1. Year 2023

In the first year of the study, we found no effect of sites on the abundance of larvae in mechanical trunk traps (df = 2, F = 2.20, *p* = 0.0752), but the abundance of captures differed significantly according to the type of trunk trap (df = 2, F = 7.07, *p* < 0.05) (Figure 9). No impact of experimental block was detected (df = 1, F = 8.05, *p* = 0.1414). There was no significant impact of interactions between major factors detected, i.e., between trap type and experimental area (df = 4, F = 15.15, *p* = 0.2017). During 2023, in Area 1, 677 larvae were caught (16 in ‘Commercial 1’, 632 larvae in ‘Prototype’, and 29 larvae in ‘Commercial 2’ traps); in Area 2, 617 larvae were caught (14 in ‘Commercial 1’, 625 in ‘Prototype’, and 32 in ‘Commercial 2’ traps). In area 3, 18 larvae were detected in ‘Commercial 1’ traps, 649 were captured in ‘Prototype’ traps, and 25 were caught in ‘Commercial 2’ traps.

#### 3.2.2. Year 2024

In the second year of the study, we found no effect of sites (areas) on the abundance of larvae in the trunk traps (df = 2, F = 3.03, *p* = 0.1412). No impact of experimental block was detected (df = 1, F = 6.55, *p* = 0.2517). No interactions between major project factors were detected, i.e., between trap type and experimental area (df = 4, F = 7.44, *p* = 0.0957).

Abundance of larvae varied by trunk trap type, with average numbers of 0.24 ± 0.06 larvae/trap/day in ‘Commercial 1’ trunk traps, 0.31 ± 0.14 larvae/trap/day in ‘Commercial 2’ trunk traps, and 0.55 ± 0.16 larvae/trap/day in ‘Prototype’ traps.

In 2024, 186 larvae were caught in area 1 (38 in ‘Commercial 1’, 102 in ‘Prototype’, and 46 in ‘Commercial 2’ traps), 189 larvae were caught in area 2 (50 in ‘Commercial 1’, 88 in ‘Prototype’, and 51 in ‘Commercial 2’ traps), and 172 larvae were caught in area 3 (38 in ‘Commercial 1’, 87 in ‘Prototype’, and 47 in ‘Commercial 2’ traps). The total number of captured spongy moth larvae varied considerably between years in the two countries. In Greece, 2040 larvae were caught in 2023 and 547 were caught the following year. In both years (2023: 50.6%; 2024: 93.4%), the highest larva captures were confirmed in the ‘Prototype’ traps, while the ‘Commercial 1’ and ‘Commercial 2’ traps did not prove effective in capturing older larvae in Greek trials.

The first larvae were recorded in the trunk traps in the third week after the setup of the experiment, when more than 1.4 larvae/trap/day was recorded in the ‘Prototype’ traps. The highest abundance of larvae was detected in the period from 10 April to 22 April 2024, with ‘Prototype’ traps catching the majority of larvae (Figure 10).

### 3.3. Weather Data from Slovenian Experimental Areas

In 2022, we monitored weather parameters from 5 May to 15 July (Table 1). In areas 1 and 2, temperatures ranged from 16.04 °C in the period 5–11 May to almost 25 °C in the period 24–30 June. Almost 63 mm of rain were recorded in the first week of June. The same week of June also had the highest number of rainy days. In area 3, an average temperature of higher than 25 °C was detected in the first week of June, and only one rainy day was detected. The highest quantity of rain, 141.30 mm, was detected on 12–18 May.

In 2023, more than 25 rainy days were detected in all areas (Table 2). The highest average daily temperatures were recorded in the beginning of July, when an average temperature higher than 23 °C was detected. In the period 11–17 May in areas 1 and 2, almost 100 mm of rain fell in six rainy days, and in area 3, around 70 mm of rain fell in just one day. In the period 14–21 June. there were no rainy days.

In 2024, Slovenian average daily temperatures ranged from 12.33 °C to 24.01 °C in all areas. The highest number of rainy days, six, was recorded in the period 28 May–10 June. (Table 3).

### 3.4. Weather Data from Greek Experimental Areas

Throughout the entire experimental period in 2023, we detected only 11 rainy days where daily precipitation exceeded 1 mm of rainfall. At the beginning of May, average daily temperatures reached 18 °C. The highest value of rain was recorded from 29 April to 5 July (Table 4).

We collected data from 26 March to 11 June 2024. During the entire experimental period, we detected only six rainy days in Greece. On the other hand, 15 rainy days were detected in Slovenia at the same period. Average daily temperatures were higher than 15 °C in all time intervals (Table 5).

### 3.5. DD Computations for Slovenia and Greece

According to the analyzed data across all three years of the Slovenian experiment, the first occurrence of old larvae occurred when ≥417 DD had accumulated. The last occurrence of old larvae occurred when ≥939.90 DD had accumulated. In 2023, DD reached 133.90 when young larvae occurred. Details are presented in Table 6.

Regarding data obtained in Greece, larvae were seen for the first time when DD was ≥595. 20. The last larvae were seen when ≥947 DD had accumulated. Details can be found in Table 7.

## 4. Discussion

In this study, we tested methods of capturing spongy moth larvae with different types of trunk traps. Research was conducted in Slovenia and Greece. This was the first study where the trap types used herein were tested for catching spongy moth larvae. Our research demonstrated that the main factors that influenced trunk trap efficacy were the local climate and the size (developmental stage) of the larvae.

Young larvae of the spongy moth started to appear in Slovenia at the end of April and older ones at the end of May, which was consistent with current findings on the pest’s bionomics in the area [32], which is characterized by the third-largest forest cover in Europe [33]. We found that the effectiveness of mechanical trunk traps for capturing larvae varied according to the shape (type) of the trap, which was also confirmed in [18]. Likewise, the capture of larvae in mechanical traps varied according to the larval developmental stage [34]. Throughout the entire investigation in Slovenia, only older larvae were trapped in ‘Commercial 1’ and ‘Commercial 2’ traps, looking for darker places (where they spend the day, as they cannot tolerate daylight) or suitable places for pupation. For younger larvae, ‘Commercial 1’ and ‘Commercial 2’ traps were not suitable in Slovenian conditions, as young larvae mostly remain in the lower canopy throughout the day [23]. In the contract, old larvae are very mobile [23]. In Slovenia, we found young larvae only in the first year of testing the ‘Prototype’ traps, when they were caught on the sticky surface of the traps in quite large numbers before moving from their oviposition sites to the canopy. The larvae were caught in the traps despite the fact that there were two rainy days on both of the first dates of their counts, although rainfall is known to reduce the effectiveness of the glue in capturing insects [35].

In contrast, in Greece, the highest number of larvae was captured in the ‘Prototype’ traps. This was attributed to the low (3–4 m) oak trees, which had lower canopy branches [36,37] much closer to the mechanical trunk traps than the canopy branches of the tall trees in Slovenia (20–30 m). The second reason for the higher efficacy of the ‘Prototype’ traps in Greece was the drier climate, with only 92 mm of rain falling in 11 rainfall days over three and a half months at the trial sites in 2023 and about half as much rain in half as many rainfall days in the year 2024. However, at the testing sites in Slovenia, rainfall between 213 and 334 mm fell in 19 to 31 rainy days over the three years, reducing the effectiveness of the adhesive in 2023 and 2024, when the ‘Prototype’ traps were also included in the trial. The negative influence of precipitation on the performance of sticky tapes has not been confirmed so far in catching caterpillars [38]; however, this has been reported for sticky traps for catching mosquitoes in an urban environment. On the other hand, it has been reported that sticky ribbons exposed to direct sun quickly lose their stickiness [39].

Temperature and rainfall are important factors influencing the presence (abundance) of pests in forests [40,41], and they are also important factors in the effectiveness of different types of traps, such as pitfall traps [42] and pyramid traps [43,44]. Young spongy moth larvae leave their egg clusters and move around the tree only when temperatures exceed 10 °C [45]. In the Slovenian areas in 2023, trunk traps were set soon enough to intercept young larvae in the ‘Prototype’ traps. Again, as mentioned in [46], temperature has an impact on the spread of harmful organisms. In Slovenian forests, the ‘Prototype’ traps were not suitable for catching late-instar larvae. On the other hand, the design of the ‘Commercial 2’ trap allowed capturing old larvae even on rainy days, as water drains faster from this trap than from the ‘Commercial 1’ trap. In the latter, during heavy rainfall, water is retained in the bag, which can cause the bag to detach from the central part of the trap and fall to the ground. Suppression of old larvae can have a greater impact, since the majority of feeding is accomplished by the late instars [47].

As mentioned above, in Greek forests, the ‘Commercial 1’ and ‘Commercial 2’ traps were less effective in capturing older larvae than the ‘Prototype’ traps.

In our study, we also attribute a major influence on the development of spongy moth larvae to temperature, which can affect pest development directly or indirectly [48]. Under Slovenian conditions, old larvae in ‘Commercial 1’ and ‘Commercial 2’ traps emerged at DD values ranging from 417 to 1166. Emergence of young larvae in the ‘Prototype’ traps was observed at a DD value of 133.90, but larvae were already found in the traps at the first inspection, which implies that they had already started to emerge earlier. In Greece, on the other hand, larvae appeared in traps at DD values of 595.20 to 1538.10.

We believe that mechanical trunk traps are suitable for capturing spongy moth larvae in urban areas where arboreal species are of high importance [49,50]. As stated in [51], urban trees and forests are important for providing ecosystem services to growing urban populations. Their biggest threat is damage by invasive insects, as insect outbreaks are more common in urban areas [51]. The severity of gipsy moth outbreaks in urban and suburban forests has resulted in costs from public and private investments in suppression [52]. Therefore, the investigation of new methods for the control of tree pests in urban environments is important from the point of view of applicability, as the use of chemical agents is particularly undesirable in such areas [53,54]. On the other hand, these traps can be accidentally removed, tampered with, or displaced by people passing by, which can compromise efficacy [55]. Therefore, several workshops [56] were organized to educate the public and maintain public awareness about new methods of gypsy moth larvae suppression.

## 5. Conclusions

Based on the results of our survey, we can conclude that in areas with a temperate climate characterized by substantial rainfall during the growing season, the ‘Commercial 2’ mechanical trunk traps were the most suitable for trapping older spongy moth larvae, as more caterpillars were caught in them than ‘Commercial 1’ traps. Other factors influencing the decision to use ‘Commercial 2’ traps include their easy setup on trunks and the ease of changing plastic containers when checking captures. ‘Commercial 2’ traps also do not contain a sticky surface, which can be an undesirable feature when using mechanical traps in climates with high rain precipitation. ‘Commercial 1’ traps in both countries caught fewer older larvae than ‘Commercial 2’ traps. Additionally, the ‘Commercial 1’ traps have a sticky inner surface, and their bags can fill with water during heavy rains; therefore the bags can fall out of the trap. Old larvae were found in both trap types even when the traps were placed on trunks without egg clusters, suggesting their usefulness even in environments with lower spongy moth abundance, where larvae also move among canopies during the growing season in denser stands. The ‘Prototype’ traps proved to be suitable for areas with regular rainfall during the growing season only for capturing young larvae during periods of low rainfall. We did not find older larvae on such traps during periods of higher rainfall, nor did we find young larvae on such traps when the traps were placed on trunks without spongy moth egg clusters. In Slovenia, regular rainfall in dense forest stands resulted in almost permanent wetness of the sticky surface of the traps, and the ‘Prototype’ traps proved unsuitable for capturing spongy moth larvae. The ‘Prototype’ and ‘Commercial 1’ traps were less user-friendly than the ‘Commercial 2’ traps because of the sticky surface of the inner part, which often remains in the user’s hands when installing the traps, changing trap parts, or counting larvae and makes further work difficult. In the ‘Prototype’ design, stickiness has to be maintained by repeated applications of glue during the period of larval emergence. In areas of lower rainfall in Greece, the ‘Prototype’ traps proved to be the most effective, as the drier climate made it easier for users to maintain the permanent stickiness of the inner part of the trap, and the lower, almost bushy oaks made it easier for the larvae to come into contact with the sticky surface of the trap even during short migrations on the trees. In suburban environments with sufficient rainfall (precipitation), ‘Commercial 2’ traps were the most suitable for capturing older spongy moth larvae, while in drier areas, the ‘Prototype’ traps were the most effective.

## Figures and Tables

**Table 1:** Weather parameters, obtained for all Slovenian experimental areas in 2022.

	AREA 1 and AREA 2				AREA 3			
Time Interval	Average Daily Temperature (°C)	Average Daily Precipitation (mm)	Sum of Precipitation (mm)	Number of Rainy Days	Average Daily Temperature (°C)	Average Daily Precipitation (mm)	Sum of Precipitation (mm)	Number of Rainy Days
5 May–11 May	16.04	2.76	19.30	4	18.16	2.33	16.30	2
12 May–18 May	20.01	0.41	2.90	2	20.18	0.46	141.30	1
19 May–27 May	19.47	3.69	33.20	2	19.96	2.98	26.80	2
28 May–02 June	15.53	3.28	19.70	3	16.20	3.42	25.20	1
3 June–09 June	20.77	8.96	62.70	5	21.33	5.87	41.10	4
10 June–16 June	20.07	0.49	3.40	2	20.32	0.20	1.40	1
17 June–23 June	21.40	0.89	6.20	2	22.11	2.54	17.80	3
24 June–30 June	24.91	4.70	32.90	1	25.84	1.57	11.00	1
1 July–06 July	23.17	4.37	26.20	1	23.45	3.42	3.42	3
7 July–15 July	19.22	0.79	7.10	1	19.52	0.54	4.90	2
Σ */SUM **	20.06 *	3.03 *	213.60 **	23 **	20.71 *	2.33 *	289.22 **	20 **

* stands for the average daily value of a specific parameter throughout experimental period (from 5 May to 15 July); ** stands for the summed value of a specific parameter throughout the experimental period (from 5 May to 15 July).

**Table 2:** Weather parameters obtained for all Slovenian experimental areas in 2023.

	AREA 1 and AREA 2				AREA 3			
Time Interval	Average Daily Temperature (°C)	Average Daily Precipitation (mm)	Sum of Precipitation (mm)	Number of Rainy Days	Average Daily Temperature (°C)	Average Daily Precipitation (mm)	Sum of Precipitation (mm)	Number of Rainy Days
20 April–26 April	12.30	1.16	8.10	2	13.16	2.26	15.80	1
27 April–4 May	12.60	1.85	14.80	2	13.28	3.19	22.50	4
5 May–10 May	14.18	0.70	4.20	1	15.23	0.98	5.90	2
11 May–17 May	11.81	13.94	97.60	6	12.40	9.84	68.90	6
18 May–26 May	17.28	1.13	10.20	1	17.89	1.08	9.70	2
27 May–1 June	18.55	0.23	1.40	1	19.02	0.23	1.10	1
2 June–8 June	17.94	1.30	9.10	2	18.44	4.96	34.70	4
9 June–13 June	17.56	5.96	29.80	3	19.02	3.12	15.60	1
14 June–21 June	21.38	0.00	0.00	0	21.59	0.00	0.00	0
22 June–28 June	21.14	5.91	41.40	3	21.47	8.13	56.90	3
29 June–5 July	20.96	2.33	16.30	3	21.51	4.03	28.20	2
6 July–11 July	23.10	0.30	1.80	1	24.00	3.20	19.40	1
12 July–19 July	23.55	6.35	50.80	4	22.08	6.94	55.50	4
Σ */SUM **	17.87 *	3.17 *	285.50 **	29 **	18.39 *	3.69 *	334.20 **	31 **

* stands for the average daily value of a specific parameter throughout the experimental period (from 20 April to 19 July); ** stands for the summed value of a specific parameter throughout the experimental period (from 20 April to 19 July).

**Table 3:** Weather parameters obtained for all Slovenian experimental areas in 2024.

	AREA 1 and AREA 2				AREA 3			
Time Interval	Average Daily Temperature (°C)	Average Daily Precipitation (mm)	Sum of Precipitation (mm)	Number of Rainy Days	Average Daily Temperature (°C)	Average Daily Precipitation (mm)	Sum of Precipitation (mm)	Number of Rainy Days
22 April–3 May	12.33	2.15	25.80	5	12.49	2.28	31.50	1
4 May–17 May	15.48	4.63	64.80	5	16.57	2.78	38.90	4
18 May–27 May	17.86	4.78	47.80	3	18.35	4.94	49.40	4
28 May–10 June	19.34	3.17	44.44	6	19.88	3.82	53.60	6
11 June–20 June	20.00	2.36	23.60	1	19.95	2.89	28.90	3
21 June–3 July	22.48	1.93	25.10	3	22.62	1.40	18.20	1
4 July–10 July	23.33	0.00	0.00	0	24.01	0.00	0.00	0
Σ */SUM **	18.69 *	2.72 *	231.54 **	23 **	19.12 *	2.59 *	220.50 **	19 **

* stands for the average daily value of a specific parameter throughout the experimental period (from 22 April to 10 July); ** stands for the summed value of a specific parameter throughout the experimental period (from 22 April to 10 July).

**Table 4:** Weather parameters obtained for all Greek experimental areas in 2023.

Time Interval	Average Daily Temperature (°C)	Average Daily Precipitation (mm)	Sum of Precipitation (mm)	Number of Rainy Days
21 April–28 April	15.72	0.12	1	0
29 April–5 May	16.63	3.66	25.60	4
6 May–12 May	18	0.06	0.40	0
13 May–19 May	20.04	0.26	1.80	1
20 May–26 May	19.74	3.06	21.40	3
27 May–2 June	21.04	3.57	25.00	2
3 June–9 June	22.48	0	0	0
10 June–23 June	23.35	1.21	17	1
24 June–30 June	25.55	0	0	0
1 July–7 July	25.86	0	0	0
Σ */SUM **	20.84 *	1.19 *	92.20 **	11 **

* stands for the average daily value of a specific parameter throughout the experimental period (from 21 April to 7 July); ** stands for the summed value of a specific parameter throughout the experimental period (from 21 April to 7 July).

**Table 5:** Weather parameters obtained for all Greek experimental areas in 2024.

Time Interval	Average Daily Temperature (°C)	Average Daily Precipitation (mm)	Sum of Precipitation (mm)	Number of Rainy Days
26 March–2 April	17.05	0.18	1.40	0
3 April–9 April	15.51	0.17	1.20	0
10 April–16 April	17.80	0.03	0.20	0
17 April–22 April	17.03	3.33	20.00	2
23 April–30 April	18.01	0.63	5.00	1
1 May–7 May	17.50	0.71	5.00	1
8 May–15 May	18.30	0.78	6.20	2
16 May–22 May	23.44	0.00	0.00	0
23 May–29 May	20.21	0.14	1	0
30 May–5 June	22.97	0.00	0	0
6 June–11 June	24.43	0.00	0	0
Σ */SUM **	19.32 *	0.54 *	40 **	6 **

* stands for the average daily value of a specific parameter throughout the experimental period (from 26 March to 11 June); ** stands for the summed value of a specific parameter throughout the experimental period (from 26 March to 11 June).

**Table 6:** DD values in Slovenian experimental areas in relation to the first and last occurrences of larvae between the years 2022 and 2024.

Year and Area	Time Interval	First/Last Occurrence	DD Value (°C)
2022			
Areas 1 and 2	19–27 May	First—OL	433.40
	24–30 June	Last—OL	898.70
Area 3	19–27 May	First—OL	479.90
	1–6 July	Last—OL	1065.10
2023			
Areas 1 and 2	20–6 April	First—YL	133.90
	27 May–1 June	First—OL	417.30
	6–11 July	Last—OL	995.00
Area 3	20–6 April	First—YL	172.60
	27 May–1 June	First—OL	480.10
	6–11 July	Last—OL	939.90
2024			
Area 1	4-17 May	First—OL	434.00
	21 June–2 July	Last—OL	1036.10
Area 2	4-17 May	First—OL	434.00
	3–10 July	Last—OL	1166.7
Area 3	18–27 May	First—OL	638.30
		Last—OL	947.40

YL = young larvae; OL = old larvae.

**Table 7:** DD values in Greek experimental areas in relation to the first and last occurrences of larvae between the years 2023 and 2024.

Year and Area	Time Interval	First/Last Occurrence	DD Value (°C)
2023			
Areas 1, 2, and 3	21–28 April	first	648.10
	24–30 June	last	1538.1
2024			
Areas 1, 2, and 3	3–9 April	first	595.20
	16–22 May	last	1098.1

## Data Availability

The original contributions presented in this study are included in the article. Further inquiries can be directed to the corresponding author.
